# Systems-Level Support for Hybrid Quantum-Classical Learning: A Systematic Review with a Medical Imaging Translation Lens

**DOI:** 10.3390/jimaging12060232

**Published:** 2026-05-28

**Authors:** Maqsudur Rahman, Pintu Chandra Paul, Amena Begum, Kashmi Sultana, Nahida Akter, Anup Majumder, Mengran Zhu, Ze Sheng, Wangjiaxuan Xin, Xin Jin, Jun Zhuang

**Affiliations:** 1 Department of Computer Science, Boise State University, Boise, ID 83725, USA; mrrajon@cou.ac.bd (M.R.);; 2 Department of Inforamtion and Communication Technology, Comilla University, Cumilla 3500, Bangladesh; 3 Department of Computer Science and Engineering, Jahangirnagar University, Savar 1342, Bangladesh; 4 Aurorie PTE Ltd., 68 Circular Road, #02-01, Singapore 118261, Singapore; 5 Department of Computer Science and Engineering, Texas A&M University, College Station, TX 77843, USA; zesheng@tamu.edu; 6 Department of Software and Information Systems, University of North Carolina at Charlotte, Charlotte, NC 28223, USA; 7 Department of Computer Science and Engineering, The Ohio State University, Columbus, OH 43210, USA

**Keywords:** hybrid quantum-classical learning, quantum machine learning, medical imaging, systematic review, runtime systems, image encoding, scheduling, memory management, reproducibility

## Abstract

Hybrid quantum-classical learning pipelines combine conventional accelerators, quantum runtimes, and quantum processing units (QPUs), creating scheduling, memory, isolation, encoding, and deployment challenges that are not captured by application-level quantum machine learning surveys alone. This paper presents a systematic review of runtime and systems mechanisms for hybrid quantum-classical workloads, with medical imaging used as a translation lens rather than as an exclusive inclusion boundary. Following a PRISMA-aligned review process, we screened 364 records and synthesized 40 studies published between 2020 and 2025. Each study was coded by systems layer, application grounding, noisy-label relevance, and evaluation maturity. The coding shows that the corpus combines direct medical evidence with broader transferable systems evidence: 8 studies directly evaluated medical data, 12 were medically motivated, and 20 were generic systems studies. Across the corpus, the strongest support concerns hybrid orchestration, qubit/resource allocation, classical–quantum data movement, and container-based reproducibility, whereas evidence remains limited for realistic clinical operation, end-to-end remote-QPU workflows, multi-tenant isolation, and noisy-label retraining loops. We contribute an evidence map, a direct/indirect/interpretive evidence distinction, and cross-layer design guidelines for future hybrid quantum-classical imaging pipelines in regulated settings.

## 1. Introduction

Medical image classification has achieved remarkable accuracy with deep learning, but these successes rely on large, accurately labeled training datasets [1]. In practice, medical labels are often noisy or inconsistent due to annotator variability and errors, which can degrade model performance [2]. Learning with noisy labels has therefore become an important research focus in medical AI [3]. A variety of algorithmic techniques—e.g., co-teaching of dual networks [4], label correction via knowledge distillation [5,6], and self-supervised learning [7,8]—have been proposed to make training more robust to mislabeled data. These methods improve accuracy under noise but typically incur additional computation (training multiple models, iterative relabeling, etc.), making training even more resource-intensive.

Recent surveys and benchmark platforms such as MedMNIST have highlighted the growing need to account for noise tolerance in realistic medical imaging datasets [3,9]. Other studies have proposed robust classification frameworks and uncertainty-aware strategies to detect or adaptively learn from label inconsistencies in medical imaging tasks [10]. These methods enhance reliability but often increase memory and computation costs, reinforcing the need for efficient resource scheduling and management.

Parallel to these developments, quantum computing has advanced to the point that quantum machine learning (QML) techniques are increasingly being explored for imaging tasks [11,12,13,14]. Quantum neural networks (QNNs), which integrate parameterized quantum circuits into neural network models, have shown promise in medical imaging applications such as retinal, X-ray, and general biomedical image classification [11,12,15]. Early studies suggest that hybrid quantum-classical models can achieve competitive performance while using compact trainable representations [13,14,16]. These developments motivate closer examination of the systems support needed to make such pipelines practical in imaging settings.

However, current quantum hardware remains constrained by noise, limited qubit counts, and device variability [17,18]. As a result, many hybrid quantum-classical models are still evaluated under simulator-based or tightly controlled conditions, and their advantages over strong classical baselines remain inconsistent [11,19,20,21]. This gap has prompted growing interest in hybrid quantum-classical approaches, where quantum components are combined with classical deep learning to balance expressiveness, hardware limitations, and practical deployability [15,16,22]. For medical imaging, this makes systems support especially important because robustness, latency, reproducibility, and secure execution all affect translational feasibility.

Running hybrid QNN workloads introduces new systems-level challenges. Unlike conventional deep learning pipelines that execute largely within mature CPU/GPU software stacks, hybrid quantum-classical workloads must coordinate classical accelerators, quantum runtimes, and specialized quantum processing units (QPUs), for which software support is still evolving [23,24,25]. Hybrid workloads require orchestrating computations across classical and quantum resources, potentially in a distributed environment (e.g., a hospital server cluster connected to a cloud QPU). The operating system must schedule tasks between classical and quantum processors, manage memory across different hardware (from RAM and GPU memory to quantum memory/qubits), ensure security of sensitive medical data and models, and provide a scalable execution environment (potentially using virtualization or containers to encapsulate the complex software stack). Each of these aspects becomes even more critical when training with noisy labels: for example, robust training might need repeated execution of certain data through the quantum model (impacting scheduling), storage of additional metadata for label noise handling (impacting memory), and careful isolation (since erroneous labels could be exploited or require special logging for audit).

This paper presents a systems-oriented systematic literature review (SLR) of runtime and systems mechanisms for hybrid quantum-classical learning. The review is motivated by medical imaging, but it does not assume that the evidence base is exclusively medical. Instead, we treat medical imaging as a demanding application lens because it combines privacy regulation, heterogeneous on-premise/cloud execution, large classical tensors, and retraining or re-validation under imperfect annotations. Throughout the paper, we therefore distinguish between *direct medical evidence* and *transferable systems evidence* from broader hybrid quantum-classical literature.

This scope clarification is important. Many mechanisms discussed in the literature—including scheduling, memory management, isolation, containerization, and runtime orchestration—are fundamentally systems questions rather than domain-specific medical innovations. The contribution of this review is thus not to claim that these mechanisms are unique to healthcare but to identify which of them are likely to matter for future quantum-enabled medical pipelines and which remain speculative under realistic operating conditions. Accordingly, the term “medical imaging translation lens” has a specific meaning in this review. We do not treat every included paper as a medical imaging study. Instead, we ask whether systems mechanisms developed in broader hybrid quantum-classical computing can be meaningfully translated to imaging workflows that involve large image tensors, privacy-sensitive data, repeated training or validation, and strict reproducibility requirements. This distinction is essential because only a subset of the current literature directly evaluates medical imaging data, whereas many scheduling, memory, security, and deployment mechanisms are evaluated in generic quantum-systems settings.

The revised review makes four contributions. First, it synthesizes the systems literature on hybrid quantum-classical scheduling, memory/data movement, isolation/security, and deployment. Second, it codes each study by evaluation maturity (conceptual, simulator-only, noisy simulation, or real hardware/hardware-in-the-loop). Third, it separates direct evidence from medical-imaging studies from cross-domain transfer arguments. Fourth, it derives a set of cross-layer design guidelines for future hybrid quantum-classical deployments in regulated environments.

Our review is guided by the following research questions (RQs):

- **RQ1 (Scheduling and orchestration):** What scheduling and orchestration mechanisms have been proposed for hybrid quantum-classical workloads?
- **RQ2 (Memory and data movement):** What memory management and data movement abstractions support efficient classical–quantum interoperability?
- **RQ3 (Isolation, security, and auditability):** What systems and runtime mechanisms are proposed to isolate workloads and protect data and models in shared hybrid quantum environments?
- **RQ4 (Virtualization and deployment):** What evidence exists on the effects of virtualization and containerization on performance, reproducibility, and scalability?
- **RQ5 (Translation to medical imaging):** Which of the above findings are directly supported in medical imaging or noisy label settings, and which remain application-level hypotheses?

To make the application lens concrete, we consider four representative medical imaging scenarios throughout the review. First, radiological image classification, such as chest X-ray or CT-based diagnosis, requires high-throughput inference, model auditability, and reliable retraining when annotation policies change. Second, digital pathology and whole-slide imaging involve extremely large tensors that cannot be directly loaded into near-term quantum circuits, making feature extraction, patching, and encoding overhead central systems concerns. Third, fundus and retinal image screening often involve privacy-sensitive distributed workflows, where local preprocessing and secure remote execution are important. Fourth, real-time or assisted diagnosis scenarios, such as triage or intraoperative support, impose stricter latency and queueing constraints than offline model development. These scenarios motivate the translation of systems mechanisms into imaging-specific requirements for training, inference, retraining, and audit. By organizing the review in this way, we aim to provide a clearer and more defensible picture of the field, one that is centered on systems evidence, explicit about evidence maturity, and careful when translating generic systems results into clinical or medical AI implications. Section 2 describes the review protocol and study-coding scheme. Section 3 first characterizes the evidence base and then synthesizes findings by research question. Section 4 positions the review relative to recent application-level surveys in digital health and medical imaging. Finally, Section 5, Section 6 and Section 8 discuss implications, limitations, and threats to validity.

## 2. Methodology

This review was conducted as a systems-oriented systematic literature review and reported using a PRISMA 2020-aligned structure [26]. Because the target literature spans systems, quantum machine learning, and medical AI, the review combines structured database search, backward snowballing, and descriptive quantitative synthesis rather than formal meta-analysis. A meta-analysis was not appropriate because the included studies differ substantially in hardware backends, simulators, workloads, datasets, performance metrics, and evaluation goals. Due to heterogeneity in evaluation setups, datasets, and hardware configurations, quantitative meta-analysis was not feasible; instead, we adopted descriptive quantitative synthesis combined with qualitative thematic analysis.

### 2.1. Review Objective and Unit of Analysis

The unit of analysis in this review is a study proposing, implementing, or evaluating a systems or runtime mechanism relevant to hybrid quantum-classical execution. We use the term *systems-level* to cover operating-system-adjacent mechanisms, runtimes, orchestrators, compiler-assisted resource managers, and deployment frameworks. Purely algorithmic QML papers were retained only when they made explicit claims about execution, resource management, deployment, or hardware-aware operation.

Medical imaging was treated as an application lens rather than a mandatory inclusion condition for every paper. This decision reflects the current state of the literature: many relevant scheduling, memory, isolation, and deployment mechanisms are proposed in general quantum-systems venues and later interpreted for medical workloads. To avoid overstating domain specificity, we explicitly coded each study for direct medical relevance and noisy-label relevance.

### 2.2. Evidence Categories

Because the corpus includes both medical and non-medical studies, we used three evidence categories throughout the synthesis. *Direct evidence* refers to studies that evaluate a medical imaging dataset, medical imaging workload, or medical QML pipeline. *Indirect evidence* refers to studies that evaluate generic hybrid quantum-classical systems mechanisms, such as scheduling or memory management, whose assumptions are transferable to imaging pipelines but not evaluated on medical data. *Interpretive synthesis* refers to implications derived by the authors when mapping systems mechanisms to medical imaging requirements such as image encoding, retraining, privacy, auditability, or clinical workflow constraints. These categories are used to avoid presenting transferable systems evidence as if it were direct medical validation.

### 2.3. Search Strategy and Sources

Searches were conducted across IEEE Xplore, Scopus, SpringerLink, ACM Digital Library, and arXiv. The final search was completed on 27 March 2026. Searches were restricted to English-language publications from 2020 to 2025. We included journal articles, conference papers, and technically detailed preprints when they provided extractable systems or runtime information. Google Scholar was used only for citation tracing and version disambiguation, not as a primary search source.

The search strategy combined three concept blocks: (i) hybrid quantum execution, (ii) systems/runtime mechanisms, and (iii) medical-imaging or annotation-noise terms. Database-specific search strings and restrictions are reported in Table 1.

Deduplication was performed using DOI, title, first author, and publication year. When both preprint and peer-reviewed versions of the same work were available, the peer-reviewed version was retained unless the preprint contained additional systems details not present in the archival version. Records with identical titles but different metadata were manually inspected.

### 2.4. Eligibility Criteria

Studies were included if they satisfied all of the following criteria:

- **Systems relevance:** The paper proposed, analyzed, or evaluated a systems/runtime mechanism for hybrid quantum-classical execution.
- **Hybrid execution relevance:** The mechanism was relevant to coordinated CPU/GPU/QPU workflows, quantum resource management, or deployment/runtime support.
- **Evidence sufficiency:** The paper contained enough technical detail to support extraction of mechanisms, assumptions, and evaluation context.

Studies were excluded if they were:

- purely algorithmic QML papers with no systems/runtime implication;
- purely hardware papers with no software/runtime interface contribution;
- cryptographic or security papers unrelated to execution environments;
- editorials, tutorials, blogs, marketing pages, or vendor notes used only as non-archival context.

A paper did *not* need to be directly medical to be included, but it was coded separately as generic, medically motivated, or directly evaluated on medical data. This distinction is central to the revised synthesis.

### 2.5. Study Selection

After deduplication, titles and abstracts were screened against the eligibility criteria. Full texts were then assessed for technical depth, systems relevance, and extractable evidence. Reasons for exclusion at the full-text stage included algorithm-only focus, hardware-only focus, insufficient systems contribution, and duplicate or superseded versions. Screening decisions were checked iteratively among the authors, and ambiguous cases were resolved through discussion.

### 2.6. Data Extraction, Coding, and Critical Appraisal

For each included study, we extracted: publication type, year, venue, application domain, systems layer, claimed contribution, evaluation setting, reported metrics, and limitations. We then coded the studies using the rubric in Table 2. The purpose of this rubric is twofold: first to make direct versus indirect evidence explicit and second to calibrate claims according to evaluation maturity.

To make the coding process more transparent without overextending the main text, Table 3 provides a condensed study-level coding overview of representative studies across the reviewed corpus. The table reports the primary system layer, evaluation maturity, medical imaging grounding, noisy label relevance, and main reported metric categories. Some studies could reasonably belong to more than one category; therefore, the table reports the dominant coding used for synthesis rather than all possible labels.

This condensed overview complements the aggregate evidence profile in Table 4. The aggregate counts were derived from the same coding dimensions, while the condensed table is intended to make the coding logic visible in the main manuscript without converting the review into a catalog of individual paper summaries. Table 5 summarizes how the reviewed systems mechanisms influence different stages of hybrid quantum-classical medical imaging pipelines, including training, inference, retraining, and auditing workflows.

### 2.7. Evidence-Strength Labels

For the cross-layer design guidelines, we used a simple evidence-strength rubric rather than a clinical certainty-of-evidence framework such as GRADE, because the review does not synthesize clinical intervention effects. A guideline was labeled *Strong* when it was supported by multiple studies with consistent findings and at least one real-hardware, hardware-in-the-loop, or deployment-oriented evaluation. A guideline was labeled *Moderate* when it was supported by multiple simulator, framework, or prototype studies but had limited real-hardware validation. A guideline was labeled *Emerging* when it was supported mainly by conceptual proposals, early prototypes, or indirect transfer evidence. These labels are intended to calibrate systems maturity, not clinical effectiveness.

### 2.8. Descriptive Quantitative Synthesis

Because the literature is heterogeneous, we complemented thematic synthesis with descriptive quantitative characterization. Specifically, we counted and reported the number and proportion of studies by evaluation maturity, application grounding, noisy-label relevance, and publication type. This quantitative evidence map addresses a limitation of the earlier manuscript, which summarized papers thematically but did not show how mature, direct, or comparable the underlying evidence actually was.

### 2.9. Quality Appraisal and Sensitivity

Quality was not inferred only from venue reputation. Instead, the appraisal emphasized evaluation realism, reproducibility of the reported setup, and the specificity of the systems claim. Studies based only on conceptual arguments or ideal simulation were retained when they contributed useful mechanisms, but their claims were interpreted conservatively in the cross-RQ synthesis.

### 2.10. Protocol and Registration

**Figure 1:**
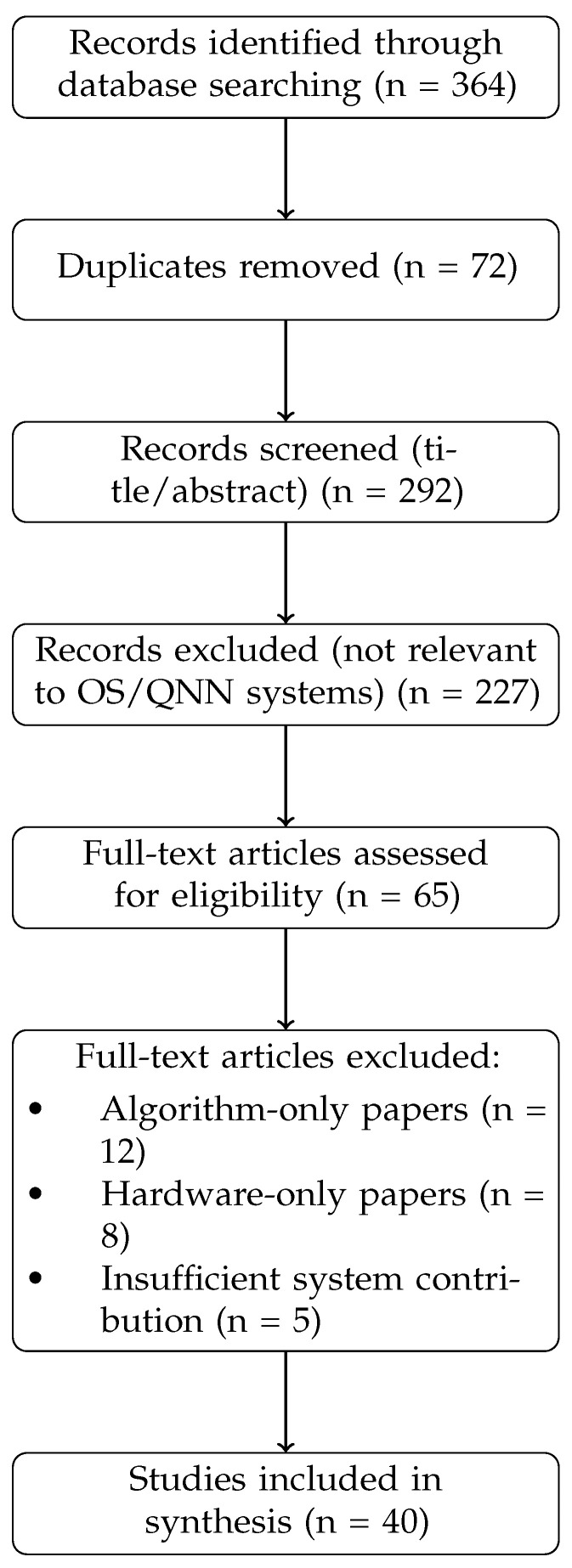
PRISMA-aligned flow diagram showing study identification, screening, eligibility, and inclusion with explicit exclusion criteria.

This systematic review was not registered in PROSPERO or any other registry. The review focuses on systems-level and runtime mechanisms for hybrid quantum-classical learning in medical imaging rather than on clinical interventions, patient outcomes, or health-effect estimates, which are the primary scope of PROSPERO registration. The review protocol was defined internally before screening and included the research questions, search strategy, eligibility criteria, and data-extraction categories. Figure 1 illustrates the PRISMA-aligned study selection workflow, including identification, screening, eligibility assessment, and final inclusion of studies used in the systematic review.

## 3. Evidence Synthesis and Cross-RQ Analysis

This section synthesizes the reviewed literature from a systems perspective and then assesses its transferability to medical imaging workloads. Because the corpus contains both direct medical studies and broader systems papers, we first characterize the evidence base itself before discussing the research questions. This ordering is deliberate: the confidence of each conclusion depends on how the underlying studies were evaluated.

### 3.1. Corpus Overview and Evidence Maturity

Before answering the individual research questions, we characterize the corpus itself. The literature is heterogeneous in three important ways: hardware realism, application grounding, and systems depth. Table 4 summarizes these distributions. This characterization serves two purposes. First, it makes clear which conclusions are directly supported by empirical evidence and which are transfer arguments from adjacent systems literature. Second, it explains why a formal performance meta-analysis was not appropriate: the studies differ substantially in hardware backends, simulators, workloads, datasets, output metrics, and experimental goals.

Overall, the corpus is weighted toward simulator-based and conceptual studies, with a smaller subset providing real-hardware or hardware-in-the-loop evidence. Likewise, only a subset evaluates medical data directly, and an even smaller subset explicitly studies noisy-label conditions. We therefore interpret performance and deployment claims conservatively throughout the remainder of the paper.

### 3.2. RQ1: Scheduling and Orchestration Mechanisms for Hybrid Quantum-Classical Workloads

Scheduling is a fundamental component of operating systems (OS), but in the context of hybrid quantum-classical workloads, it presents unique complexities. Unlike traditional workloads, hybrid quantum systems require coordinated execution between classical processors (CPUs/GPUs) and quantum processing units (QPUs), with considerations for decoherence windows, quantum noise, and circuit fidelity. Therefore, the OS scheduler must not only manage resource utilization but also address temporal constraints and noise-aware job placement, especially in the presence of competing workloads or distributed quantum infrastructures [27].

**HPC and Slurm-based Hybrid Scheduling:** In one of the earliest studies on integrated quantum-classical scheduling, Esposito et al. [27] applied the Slurm workload manager to orchestrate hybrid jobs. Their approach leverages the MPMD model to combine classical simulations and quantum circuits within a single Slurm job, managing dependencies through message passing. Although their experiment used a simulator backend, the principles are transferrable to cloud or real QPU deployments. By decoupling classical pre/post-processing and overlapping quantum evaluations, Slurm-style scheduling can minimize idle periods and reduce runtime.

**Quantum Multi-Tenancy and Runtime Management:** Extending beyond HPC environments, the idea of treating QPUs as shared, schedulable resources is gaining traction. Murali et al. [28] proposed an early framework for multi-programming on NISQ devices, showing that multiple jobs could be time-sliced across qubit subsets. This work was extended by Giortamis et al. [23], who introduced the QOS runtime, a stack that supports spatial and temporal quantum job multiplexing. QOS includes fidelity-aware and load-aware scheduling logic, which significantly reduced waiting time (up to 5×) while maintaining high accuracy. Such runtimes resemble classical OS designs where multiple user programs are isolated and multiplexed using schedulers.

**Entropy-aware and Real-Time Scheduling:** In latency-sensitive settings, such as medical QNN applications with real-time constraints (e.g., surgical diagnostics), scheduling policies must handle quantum jobs with bounded response time. Zirak [29] introduced XIRAC-Q, which uses Shannon entropy to model system capacity under quantum noise. XIRAC-Q provides a real-time scheduler that maximizes task throughput within an entropy budget, ensuring system reliability under quantum decoherence. While theoretical, it aligns with OS-level QoS (Quality of Service) strategies used in embedded and real-time systems.

**Pipeline and Hybrid-Co-Scheduling:** Modern hybrid QNN frameworks must manage alternating classical and quantum phases. This requires pipelining quantum kernel calls with classical preprocessing (e.g., feature extraction or gradient updates). The OS must provide low-latency event handling and thread management to facilitate such hybrid pipelines. Liu et al. [24] proposed a qubit partitioning-based scheduler in their QuCloud architecture, which compiles and schedules concurrent programs based on predicted error profiles. It serves as a compilation-aware OS scheduler for quantum clouds.

**Cross-Architecture Scheduling Considerations:** To generalize these ideas, emerging QNN models—such as hybrid CNN-QCNN [39] and quantum transfer learning models [14]—introduce diverse workload patterns requiring adaptive scheduling. Studies like Bokhan et al. [13] and Mazher et al. [15] emphasized workload variability based on model depth and input modality. Scheduling these pipelines requires heterogeneous policy support for small circuits (e.g., QCNN layers) and large batch training (e.g., quantum-enhanced classifiers). Trochun et al. [40] showed that even small quantum convolution layers introduce scheduling dependencies that classical OS schedulers are not optimized to manage. Therefore, advanced policies that integrate quantum job profiling, fidelity prediction, and scheduling hints are essential for scalability. Table 6 summarizes representative scheduling and orchestration mechanisms proposed for hybrid quantum-classical workloads, highlighting their main system-level objectives and operational characteristics.

**Evidence interpretation and medical transfer.** The literature reviewed under RQ1 shows stronger support for hybrid orchestration and QPU multiplexing as general systems abstractions than for end-to-end latency improvements in clinical deployments. In remote-QPU scenarios, communication, provider queueing, circuit compilation, and shot execution may dominate local scheduler overhead. Consequently, systems-level scheduling should be interpreted as most beneficial for batching, overlap, data staging, retry control, and simulator/GPU phases rather than as the sole determinant of end-to-end clinical responsiveness. For medical imaging, the practical implication is that scheduler design matters most when quantum calls are frequent, batched, or tightly coupled to classical preprocessing pipelines.

Table 7 shows that the most deployment-relevant studies report different metrics and are not directly comparable. In particular, latency, throughput, fidelity, and medical task performance are rarely reported together. This lack of common reporting is itself a key finding of the review and motivates the cross-layer reporting recommendations in Section 5.

### 3.3. RQ2: Memory and Data-Movement Mechanisms for Quantum-Classical Interoperability

Memory management in hybrid quantum-classical systems spans two tightly coupled layers: classical memory (e.g., RAM, cache, GPU memory) and quantum memory (qubits and their quantum states). Unlike classical memory, quantum memory is volatile, non-copyable (due to the no-cloning theorem [44]), and constrained in size. Consequently, OS-level mechanisms for memory allocation, reuse, data caching, and inter-device communication play a vital role in efficient execution of QNN workloads. Moreover, noise-robust training techniques such as co-teaching [4], dual-uncertainty learning, and ensemble denoising [45] often require auxiliary memory for storing intermediate representations, noise indices, or multiple model copies.

**Virtual Quantum Memory Abstractions:** Classical OSes use virtual memory to abstract hardware limitations and enforce process isolation. A similar abstraction is emerging for quantum programs. Van der Vecht et al. [30] introduced a virtual quantum memory space (VQMS) in Qoala, where quantum processes reference logical qubit indices that the OS dynamically maps to physical hardware. The QNodeOS/Qoala framework further integrates per-process memory tracking and isolation [46], providing a multi-user quantum OS analogous to classical MMU behavior.

**Efficient Qubit Recycling and Ancilla Management:** In quantum circuits, qubit reuse can dramatically reduce physical qubit requirements. Reichental et al. [33] developed a topological traversal and allocation strategy that maximizes qubit reuse based on dependency graphs. This is complemented by Ding and Chong’s work on qubit uncomputation [31], where ancilla qubits are reset and re-used within the same execution. Uncomputation (also called garbage collection in quantum logic) mimics classical deallocation, enabling OS memory manager to release qubits safely during runtime [32].

**Compiler-Assisted Memory Coordination:** Several studies bridge compiler-level optimizations with OS memory policy. Above all, Bokhan et al. [13] showed how shallow QCNN architectures reduce qubit pressure via design-time optimization. OS-level memory managers can leverage these compiler hints to reserve memory aligned with circuit topology.

**Memory in Noise-Robust Training:** Memory requirements grow in robust QNN training. Techniques like CleanNet [47], Meta-Weight-Net [30], and DUAL store confidence scores, reweighting factors, or clean/noisy sample buffers. For quantum extensions, Jiang et al. [42] proposed self-supervised learning for label noise, requiring persistent memory for tracking representation consistency. OS-level support for shared memory or pinning critical data (e.g., noise-prone samples) in fast-access RAM could reduce I/O overhead. In large medical imaging pipelines, these strategies are especially relevant because repeated feature extraction, relabeling, or consistency tracking can amplify memory pressure.

**Shared Memory and Classical-Quantum IO Optimization:** Qoala introduces a shared memory abstraction that minimizes context-switching between CPU and QPU [30]. In practical QNN workflows, input batches (e.g., images or embeddings) must be transferred repeatedly across classical and quantum memory boundaries. Traditional APIs that serialize these calls are inefficient. Instead, mapped memory buffers or zero-copy DMA channels (inspired by GPU–CPU interactions) can accelerate such pipelines. Hybrid training frameworks like Mazher et al. [15] show that prefetching and in-memory caching reduce overall latency.

#### Image Encoding and Data Movement Bottlenecks

A medical-imaging-specific bottleneck is the conversion of large image tensors into quantum circuit inputs. Radiological volumes, whole-slide pathology images, and multi-channel MRI scans can contain orders of magnitude more pixels or voxels than can be represented directly on near-term quantum devices. Therefore, encoding is not only a modeling choice but also a systems and data movement issue.

Angle encoding is relatively simple and compatible with near-term circuits, but it typically requires dimensionality reduction, patching, or feature extraction when the number of image features exceeds the available qubit count. Amplitude encoding can represent a length-*d* normalized vector using $\left\lceil \log_{2}d\right\rceil$ qubits, but generic state preparation may require circuits whose depth grows with the input dimension, making the encoding step a potential I/O and compilation bottleneck. Block encoding is theoretically powerful for representing matrices or linear operators, but it usually assumes oracle-like access or additional circuit structure that is difficult to realize in near-term medical imaging workflows.

For these reasons, most practical hybrid quantum-classical imaging pipelines are likely to use classical feature extraction, patch-level embeddings, principal component analysis, or learned compression before quantum encoding. From a systems perspective, this shifts the bottleneck from raw image transfer to feature provenance, encoding reproducibility, shot allocation, and repeated circuit invocation. RQ2 therefore treats image encoding, feature caching, and classical–quantum transfer as coupled memory management problems rather than separate algorithmic details.

**Emerging Memory Allocation Policies:** Few studies address runtime allocation under memory pressure. Liu et al.’s QuCloud [24] prioritizes “healthier” qubits (based on calibration data), effectively modeling memory allocation based on reliability—a form of noise-aware memory policy. Others like Trochun et al. [40] and Gao et al. [48] support lightweight circuits that can fit in constrained QPUs, but this reduction in qubit usage often comes at the cost of increased circuit depth, highlighting the need to analyze trade-offs between spatial (qubit count) and temporal (circuit depth) resource allocation.

**Evidence interpretation and medical transfer.** RQ2 is supported mainly by generic quantum systems papers and only secondarily by medical imaging pipelines. The most defensible conclusion is that memory efficiency is a cross-domain bottleneck: logical-to-physical qubit mapping, reuse of ancilla qubits, and reduced copy overhead are useful regardless of application. By contrast, claims about noisy-label training are presently indirect. Existing systems papers rarely measure memory behavior under co-teaching, relabeling, or uncertainty-aware retraining loops on quantum hardware. We therefore treat noisy-label medical training as a workload requirement that motivates future evaluation rather than as an empirically established use case. Table 8 summarizes representative memory, encoding, and data-movement mechanisms discussed in the literature and their relevance to efficient hybrid quantum-classical execution and medical imaging workflows.

### 3.4. RQ3: Isolation, Security, and Privacy Mechanisms for Hybrid Quantum-Classical Workloads

Security and privacy are critical in quantum-enhanced medical applications due to sensitive patient data, regulatory compliance, and the risks posed by multi-user computing environments [36,37]. Quantum operating systems (OS) must secure both the quantum and classical components of hybrid workloads while accounting for novel threat vectors such as quantum crosstalk, side-channel leakage, and circuit-level model extraction [34,35].

**Multi-Tenancy and Crosstalk Risks:** Quantum cloud platforms (e.g., IBM Quantum, AWS Braket) allow multiple users to execute circuits concurrently on shared hardware. Crosstalk between adjacent qubits can introduce exploitable leakage. Demonstrations by Ash-Saki et al. [34] and Choudhury et al. [35] showed that adversarial circuits using SWAP gates could influence or infer properties of neighboring jobs. This reveals a fundamental limitation in logical qubit isolation. Enhancing OS-level defenses—such as randomized physical qubit mapping, crosstalk-aware scheduling, and per-job fidelity audits—is essential to prevent such leakage.

**Process Isolation and Quantum Sandboxing:** Similar to classical OS process isolation, quantum systems must prevent one process from corrupting or observing another’s quantum state. Quantum sandboxes and secure runtime environments (e.g., Qiskit Runtime) already enforce limited I/O per job. Liu et al.’s QuCloud [24] demonstrates circuit partitioning for process separation, while hybrid schedulers [23] can queue jobs such that high-priority, sensitive tasks are executed in a dedicated coherence window. These policies are analogous to privilege levels or security domains in classical OS kernels.

**Secure Data Movement and Blind Computation:** From a data privacy perspective, transferring patient images, embeddings, or labels to a quantum cloud requires encryption in transit and possibly at rest. Conventional mechanisms such as TLS are necessary but insufficient if the server itself is untrusted. Quantum blind computing offers a privacy-preserving alternative: clients encode data using entangled states, and servers evaluate the circuit without learning the input/output [41]. While current blind quantum protocols are limited to small-scale systems, integration with OS I/O layers may enable practical adoption in the future.

**Circuit Confidentiality and Model Extraction Attacks:** Another vector is the reverse engineering of QNN models submitted by users. Wang et al. [7] simulated an attack where co-teaching and noise-aware techniques were used to approximate a victim QNN’s behavior through repeated API queries. This type of attack is comparable to classical membership inference, and suggests OS-level rate-limiting, quantum API obfuscation, or query-based noise injection as countermeasures.

**Runtime Monitoring and Anomaly Detection:** To enhance trust in multi-tenant quantum environments, the operating system must support runtime monitoring for anomalous or potentially adversarial circuit behavior. Recent perspective pieces have discussed early-stage proposals for integrating job-pattern inspection into the quantum software stack to identify suspicious circuit behavior and resource misuse [49]. As memory-aware QNNs become more prevalent [42], adversarial circuits may increasingly mimic benign access patterns, thus reinforcing the need for behavior-aware anomaly detection at the system level.

**Compliance and Audit Trails:** Healthcare environments must enforce data access control, auditing, and anonymization policies. The OS should generate logs of QNN executions with job ID, circuit hash, timestamps, and resource utilization—without storing raw medical data. Local preprocessing (e.g., using a classical encoder [14]) can anonymize inputs before uploading quantum kernels. Regulatory features should align with GDPR, HIPAA, and quantum-safe best practices [49].

**Quantum-specific versus conventional controls.** Security requirements in hospital-to-cloud hybrid workflows arise from two different sources. Conventional distributed-systems requirements include transport encryption, identity and access management, audit logging, consent tracking, and data minimization. Quantum-specific requirements arise from shared-QPU execution, including crosstalk, malicious circuits, qubit co-location, and calibration-dependent leakage. Distinguishing these layers is essential: the reviewed literature offers early evidence for the second class, whereas the first class largely reuses established cloud-security practice. Table 9 summarizes this distinction.

### 3.5. RQ4: Virtualization and Containerization for Performance, Reproducibility, and Scalability

Virtualization and containerization have emerged as foundational technologies in modern computing for enabling scalable, portable, and resource-efficient software deployment. In the context of hybrid quantum-classical neural networks (QNNs), these approaches are especially valuable. They facilitate encapsulation of quantum SDKs, ML libraries, and runtime environments across heterogeneous infrastructures (e.g., local clusters, edge devices, and quantum clouds). For QNN pipelines, which require precise orchestration of classical pre/post-processing with quantum kernel execution, OS-level support for virtualization is essential to optimize latency, utilization, and job distribution [25]. A summary of virtualization and containerization approaches, along with their effects on the performance and scalability of QNNs, is presented in Table 10. Table 11 summarizes the major isolation, security, and auditability considerations identified in the reviewed literature for hybrid quantum-classical systems and medical imaging workflows.

**Containerized QNN Execution Pipelines:** Quantum container orchestration has been investigated in projects like Qubernetes [25], which integrated Kubernetes primitives with quantum workflows. Tasks such as circuit preprocessing, inference scheduling, and hybrid training were mapped to containerized services, enabling elasticity and replication. This model suggests a possible deployment pattern for future hospital-linked workflows, where classical encoders, simulators, and quantum runtime services may be separated into reproducible components. However, current evidence does not yet establish end-to-end clinical scalability. Similarly, Mazher et al. [15] showed that hybrid pipelines executed in Docker containers reduced deployment friction and allowed workload balancing across GPU and QPU nodes.

**Logical Qubit Partitioning:** These ideas extend to lightweight deployments such as those proposed by Trochun et al. [40], where quantum image classifiers run on low-depth QCNNs that are well suited to containerized inference workloads. In such settings, quantum virtual machines (qVMs) can be provisioned by a hypervisor that performs compile-time scheduling to avoid real-time context switching. These systems virtualize qubit allocations, improving utilization and supporting tenant isolation in multi-user environments.

**Simulation and GPU-Backed Development Environments:** In practice, many QNN training pipelines must be validated using classical simulators due to limited QPU availability. Chen et al. [38] demonstrated that containerized simulation environments using cuQuantum achieved over 10× speedup when training hybrid convolutional networks. These setups allow reproducible and scalable training without access to dedicated quantum hardware, helping teams prototype noise-aware QNNs [42,50].

**Latency and Overhead Analysis:** Gao et al. [48] and Veit et al. [51] showed that robust models with dynamic input flow can exacerbate latency if container orchestration is not optimized. For latency-sensitive medical imaging workflows, virtualization and containerization should be evaluated together with queueing, circuit compilation, backend availability, and runtime placement, rather than treated as isolated local overheads. Furthermore, batching strategies like those in Bokhan et al. [13] reduce invocation latency by grouping circuits.

#### VQA Roundtrip Overhead and Deployment Boundary Conditions

In variational quantum algorithm (VQA)-style QNN training, the dominant deployment cost is often not a single container invocation but the accumulation of many classical–quantum roundtrips. If $N_{e}$ denotes the number of epochs, $N_{b}$ the number of batches per epoch, $N_{\theta }$ the number of circuit evaluations required for parameter updates, and $t_{o}$ the orchestration overhead per circuit call, then the accumulated orchestration cost can be approximated as

$$T_{\mathrm{overhead}}\approx N_{e}N_{b}N_{\theta }t_{o}.$$

Thus, an overhead that appears small for a single invocation can become a major component of training time when thousands of circuit calls are required. This distinction is important for medical imaging QML, where repeated retraining, validation, and uncertainty estimation may further increase the number of circuit evaluations.

The implication is that containerization should not be evaluated only through generic cloud-native overhead rules. Co-located persistent runtimes, batched circuit submission, circuit caching, and asynchronous execution may reduce repeated call overhead, whereas remote cloud-QPU workflows may be dominated by queueing, compilation, shot execution, and data return latency. Therefore, the benefit of containerization in this review is interpreted primarily as reproducibility and dependency isolation unless latency is explicitly measured in a hybrid QNN loop.

**Security, Portability, and Ecosystem Maturity:** Containerized QNN services support version control, isolation, and deployment reproducibility. Liu et al. [39] argued that packaging hybrid networks as containerized services improved ecosystem compatibility and enabled runtime swapping between quantum simulators and hardware. Jiang et al. [42] and Khanal et al. [43] highlighted that robust QNN pipelines often require fast iteration, which containers facilitate by maintaining reproducible environments for training and inference under noise.

**Evidence interpretation and deployment boundary conditions.** The literature provides stronger evidence for containers as mechanisms for reproducibility, packaging, and environment isolation than for fine-grained performance acceleration. Evidence on qVMs and hypervisor-style virtualization remains limited and often conceptual. Moreover, when hybrid control loops are latency-sensitive, container orchestration must be combined with CPU pinning, dedicated runtimes, or locality-aware deployment. Therefore, this review treats containerization and virtualization as distinct deployment strategies rather than interchangeable terms, and interprets their benefits primarily in terms of reproducibility and manageability unless direct latency measurements are reported.

### 3.6. Cross-RQ Synthesis: Design Guidelines for Hybrid Quantum-Classical Medical Pipelines

The main value of the reviewed literature is not a definitive proof of deployability but a set of recurring systems design principles. Across scheduling, memory, security, and deployment, the same cross-layer pattern emerges: hybrid quantum-classical pipelines should treat the QPU as a scarce and asynchronous accelerator, minimize unnecessary data movement, separate conventional cloud-security controls from quantum-specific isolation mechanisms, and report evaluation realism explicitly. Table 12 summarizes the resulting design guidelines.

## 4. Related Work

Recent application-level reviews provide important context for this study. Gupta et al. [19] performed a systematic review of quantum machine learning for digital health and concluded that the evidence base remains weak under realistic operating conditions. Shahriyar and Tanbhir [20] reviewed quantum machine learning for medical image classification, emphasizing the transition from simulation-only evaluation toward real quantum hardware. Idzikowski et al. [21] surveyed quantum machine learning applications in medicine and healthcare and highlighted the continued dominance of simulator-based experiments, small datasets, and inconsistent reporting.

These surveys are highly relevant, but they answer a different question from the one addressed here. Their primary focus is application-level performance, medical domain coverage, or algorithm taxonomy. They do not systematically synthesize the systems and runtime mechanisms required to execute hybrid quantum-classical workloads, nor do they explicitly separate direct medical evidence from transferable systems evidence. Our review complements rather than duplicates that literature by focusing on scheduling/orchestration, memory/data movement, isolation/security, and deployment mechanisms.

To make this distinction explicit, Table 13 compares the scope of recent surveys with the present review.

## 5. Implications and Research Agenda

The revised evidence synthesis suggests three overall conclusions. First, the current literature offers useful systems abstractions—hybrid orchestration, qubit-aware allocation, isolation policies, and reproducible deployment practices—but only limited end-to-end evidence under realistic clinical operating conditions. Second, the medical imaging connection is strongest as a workload lens that stresses privacy, data movement, and retraining requirements; it is weaker as a direct empirical corpus. Third, the main barrier to stronger claims is not the absence of proposed mechanisms, but the lack of evaluation realism and reporting consistency.

This leads to a more focused research agenda. A first priority is *evaluation realism*: future studies should report whether results come from ideal simulation, noisy simulation, hardware-in-the-loop testing, or real QPU execution, and should describe queueing assumptions, backend calibration context, and communication paths. A second priority is *cross-layer reporting*: papers should report systems metrics such as latency, throughput, memory pressure, and orchestration overhead alongside task-level performance metrics. A third priority is *secure shared execution*: as multi-tenant quantum infrastructure matures, isolation and auditability must be treated as first-class runtime concerns rather than afterthoughts. A fourth priority is *application-aware benchmarking*: medical imaging studies should explicitly measure how privacy constraints, large input tensors, label quality, and retraining loops affect the viability of hybrid quantum-classical execution.

Taken together, these points suggest that the near-term value of systems research in this area lies less in claiming immediate clinical deployment and more in establishing the runtime, reporting, and benchmarking foundations needed for future deployability.

## 6. Limitations

This review has several limitations. First, the literature itself is immature: a substantial portion of the corpus consists of preprints, simulator-based studies, or conceptual proposals. We retained these papers when they contributed useful systems ideas, but interpreted their deployment claims conservatively.

Second, the revised paper intentionally distinguishes direct medical evidence from transferable systems evidence. This is a strength analytically, but it also means that some implications for medical imaging are based on structured transfer rather than direct clinical or medical-imaging evaluation.

Third, the evidence base is highly heterogeneous with respect to hardware backends, simulators, datasets, metrics, and workload definitions. For that reason, we did not perform a formal meta-analysis. Instead, we used descriptive quantitative characterization and qualitative synthesis.

Fourth, although the revised review uses medical imaging as an application lens, the conclusions should not be interpreted as proof of clinical readiness or empirical quantum utility. They are best understood as evidence-calibrated systems implications for future hybrid quantum-classical pipeline design.

## 7. Conclusions

This review does not establish that hybrid quantum-classical medical imaging is ready for clinical deployment, nor does it establish empirical quantum utility in this domain. Instead, it synthesizes the systems-level evidence that must be addressed before such deployment claims can be made responsibly. The reviewed literature shows useful progress in hybrid orchestration, qubit-aware allocation, data-movement abstractions, security mechanisms, and reproducible deployment, but the evidence remains uneven and often indirect for medical imaging.

By repositioning the paper as a systems-level review with a medical imaging translation lens, we distinguish direct medical evidence from transferable systems evidence and author-derived interpretation. The resulting synthesis highlights concrete medical imaging requirements, including encoding large image tensors, managing repeated VQA-style circuit calls, preserving backend and compilation provenance, and supporting retraining and audit under noisy annotations. Future work should therefore move beyond isolated simulator demonstrations and report end-to-end imaging workflows with latency, throughput, fidelity, shot budget, encoding cost, and reproducibility metadata.

## 8. Threats to Validity

This section outlines methodological risks and potential biases that may affect the internal and external validity of our systematic literature review (SLR). While our review follows established best practices, some threats are inherent in the design and execution of SLRs, especially in fast-evolving domains like quantum computing.

**Search and Selection Bias:** Although we designed comprehensive search queries across IEEE, ACM, Scopus, Springer, and arXiv, the risk of missing relevant studies remains. Terminological variations (e.g., “quantum OS,” “quantum runtime,” or “scheduler”) may have caused some relevant works to be overlooked. We mitigated this via keyword expansion, boolean variations, and backward snowballing, but recall cannot be fully guaranteed.

**Publication Bias:** A substantial portion of the literature in quantum-classical OS research exists in preprint form (e.g., arXiv), with varying levels of peer review. To reduce bias, we emphasized technically detailed and frequently cited preprints, but some included works may still lack formal validation, potentially influencing our synthesis.

**Data Extraction Subjectivity:** While thematic coding and classification of papers were guided by predefined RQs and categories (scheduling, memory, security, virtualization), interpretation of some architectural features or evaluation claims may vary between reviewers. We reduced subjectivity by cross-checking extracted data, but inter-reviewer agreement was not formally computed.

**Incomplete Evaluation Coverage:** Several frameworks (e.g., QuCloud, QOS, Qoala) discussed in our synthesis are either conceptual or validated in simulated settings. This limits our ability to independently assess claims about performance, scalability, or robustness under real-world quantum hardware constraints.

**Scope-transfer threat:** Because many included studies are generic quantum systems papers rather than direct medical imaging evaluations, some medical implications in this review are transfer arguments. We mitigated this threat by explicitly coding application grounding and by distinguishing direct evidence from indirect relevance throughout the synthesis.

**Evidence-maturity threat:** A large share of the literature is conceptual or simulator-based. Such studies are valuable for identifying mechanisms, but they may overestimate deployability under real-world quantum hardware constraints. We addressed this by coding evaluation maturity and by calibrating our conclusions accordingly. 

## Figures and Tables

**Table 1:** Database-specific search strings and restrictions used for the systematic review.

Database	Search String	Restrictions
IEEE Xplore	(“hybrid quantum-classical” OR “quantum neural network” OR “quantum machine learning” OR “quantum runtime” OR “quantum operating system”) AND (scheduling OR orchestration OR “memory management” OR security OR privacy OR isolation OR virtualization OR containerization OR deployment) AND (“medical imaging” OR “medical image classification” OR healthcare OR clinical OR “noisy label” OR “annotation noise”)	English; 2020–2025; journal and conference records
Scopus	TITLE-ABS-KEY((“hybrid quantum-classical” OR “quantum neural network” OR “quantum machine learning” OR “quantum runtime” OR “quantum operating system”) AND (scheduling OR orchestration OR “memory management” OR security OR privacy OR isolation OR virtualization OR containerization OR deployment) AND (“medical imaging” OR “medical image classification” OR healthcare OR clinical OR “noisy label” OR “annotation noise”))	English; 2020–2025; articles and conference papers
SpringerLink	(“hybrid quantum-classical” OR “quantum neural network” OR “quantum machine learning” OR “quantum computing”) AND (runtime OR scheduling OR memory OR security OR virtualization OR deployment) AND (medical OR imaging OR healthcare OR noisy labels)	English; 2020–2025; research and review records
ACM Digital Library	(“hybrid quantum-classical” OR “quantum runtime” OR “quantum operating system” OR “quantum computing”) AND (scheduler OR scheduling OR memory OR security OR isolation OR virtualization OR containerization OR deployment)	English; 2020–2025; computing and systems records
arXiv	(“hybrid quantum-classical” OR “quantum neural network” OR “quantum runtime” OR “quantum operating system”) AND (scheduling OR memory OR security OR virtualization OR deployment OR medical imaging OR noisy labels)	English; 2020–2025; preprints screened for technical depth

**Table 2:** Study coding rubric for evidence characterization.

Dimension	Codes	Interpretation
Systems layer	S1: scheduling; S2: memory; S3: security; S4: deployment; S5: cross-layer	Identifies primary system-level contribution
Evaluation maturity	M0: conceptual; M1: simulator; M2: noisy/hybrid; M3: real QPU	Indicates realism of evaluation and deployment confidence
Application grounding	A0: generic; A1: medical motivation; A2: direct medical use	Distinguishes direct vs. transferable evidence
Noisy label relevance	N0: none; N1: indirect; N2: direct	Captures relevance to noisy-label training
Publication type	P0: peer-reviewed; P1: preprint	Supports cautious interpretation of results
Reported outcomes	latency, throughput, fidelity, accuracy/AUC, memory, overhead, security, reproducibility	Metrics reported; not directly comparable across studies

**Table 3:** Condensed study-level coding overview of the reviewed corpus.

Study/Group	Primary System Layer	Maturity	Medical Grounding	Noisy-Label Relevance	Main Reported Metric Category
Esposito et al. [27]	Scheduling/orchestration	Simulator/HPC	Generic	None	Runtime, job orchestration
Giortamis et al. [23]	Quantum runtime/scheduling	Real-QPU oriented	Generic	None	Waiting time, fidelity-aware scheduling
Murali et al. [28]	Multi-programming/crosstalk	Hardware-aware	Generic	None	Fidelity, crosstalk, concurrent execution
Liu et al. [24]	Qubit mapping/scheduling	Hardware-aware simulation	Generic	None	Mapping quality, fidelity estimates
Zirak [29]	Real-time scheduling	Conceptual/analytical	Generic	None	Throughput, entropy budget
van der Vecht et al. [30]	Quantum memory/runtime	Prototype/framework	Generic	None	Memory abstraction, process isolation
Ding and Chong [31]; SQUARE [32]	Qubit reuse/uncomputation	Conceptual/compiler-level	Generic	None	Qubit count, circuit resource use
Reichental et al. [33]	Qubit recycling	Framework/simulation	Generic	None	Qubit reuse, memory reduction
Ash-Saki et al. [34]; Choudhury et al. [35]	Security/crosstalk isolation	Hardware-aware/security evaluation	Generic	None	Crosstalk leakage, fidelity impact
Wang et al. [36,37]	QML security/privacy	Prototype/simulation	Generic	Indirect	Accuracy, privacy/security behavior
Stirbu et al. [25]	Container orchestration	Prototype/framework	Generic	None	Deployment, reproducibility, orchestration
Chen et al. [38]	GPU-backed quantum simulation	Hybrid simulation	Generic	None	Throughput, simulation speedup
Liu et al. [39]; Bokhan et al. [13]	Hybrid QNN architecture	Simulation	Medical motivation	Indirect	Accuracy, loss, circuit size
Dhara et al. [14]; Trochun et al. [40]	Quantum transfer/image QNNs	Simulation	Medical or imaging motivation	Indirect	Accuracy, model performance
Landman et al. [11]; Mathur et al. [41]	Medical QML evaluation	Medical QML benchmark	Direct medical	Indirect	Accuracy, classification performance
Mazher et al. [15]; Ajlouni et al. [12]	Hybrid medical QNNs	Medical imaging evaluation	Direct medical	Indirect	Accuracy, diagnostic classification
Karimi et al. [1]; Shi et al. [3]	Noisy-label medical imaging	Review/synthesis	Direct medical	Direct	Robustness methods, dataset noise
Ju et al. [10]	Uncertainty-aware noisy-label learning	Medical imaging evaluation	Direct medical	Direct	Accuracy, uncertainty, robustness
Jiang et al. [42]; Khanal et al. [43]	Robust medical image learning	Simulation/empirical	Direct medical	Direct	Accuracy, robustness to label noise
Gupta et al. [19]; Shahriyar and Tanbhir [20]; Idzikowski et al. [21]	Application-level QML review	Review/synthesis	Medical motivation	Indirect	Evidence maturity, application trends

**Table 4:** Evidence profile of the reviewed literature based on dominant coding categories. Counts are reported as synthesis-level coding summaries rather than meta-analytic estimates.

Dimension	Category	Count	Share (%)
Evaluation maturity	Conceptual/framework only	10	25%
	Simulator-based evaluation	18	45%
	Noisy simulation/hybrid testing	8	20%
	Real QPU/hardware-in-loop	4	10%
Application grounding	Generic (non-medical)	20	50%
	Medical motivation only	12	30%
	Direct medical evaluation	8	20%
Noisy-label relevance	Not addressed	22	55%
	Indirect implication	12	30%
	Direct evaluation	6	15%
Publication type	Peer-reviewed (journal/conference)	26	65%
	Preprint/emerging work	14	35%

**Table 5:** Systems mechanisms and their effects across medical imaging QML pipeline stages.

Mechanism	Training	Inference	Retraining/Validation	Auditing
Scheduling/orchestration	Batches repeated circuit calls and overlaps classical preprocessing with quantum evaluation	Controls queueing and backend selection for time-sensitive cases	Supports repeated validation under updated labels or protocols	Records job order, backend use, and failed/retried runs
Memory/data movement	Manages feature tensors, encoded inputs, and repeated gradient evaluations	Reduces transfer of large images by using local feature extraction	Stores embeddings, label-confidence scores, and retraining metadata	Preserves provenance of preprocessing, encoding, and feature versions
Security/isolation	Protects training data, labels, and model parameters in shared workflows	Limits leakage during remote quantum execution	Controls access to updated labels and retraining logs	Supports compliance logs, circuit hashes, and backend records
Containerization/deployment	Packages SDKs, simulators, and training dependencies reproducibly	Supports portable inference services across local and cloud resources	Enables reproducible retraining under fixed software environments	Documents software versions and runtime configurations

**Table 6:** Representative scheduling and orchestration mechanisms for hybrid quantum-classical workloads (RQ1).

Approach	Summary and Key Features
Slurm HPC Integration [27]	Uses Slurm to orchestrate hybrid jobs (MPMD style). Enables MPI coordination between quantum and classical sub-tasks. Proven latency savings via interleaved execution.
Quantum OS Runtime (QOS) [23]	Multi-programming runtime that spatially and temporally multiplexes quantum jobs. Incorporates fidelity/load-aware logic. Validated on IBM Q cloud.
XIRAC-Q Scheduler [29]	Entropy-based real-time quantum job scheduler. Targets latency-bound scenarios. Maximizes task throughput under system entropy budget.
QuCloud Scheduling [24]	Compiles and allocates quantum programs based on circuit error profiles. Partitions qubits by community detection and schedules with fidelity estimates.
Hybrid CNN-QCNN Schedulers [39]	Schedules layered pipelines (CNN+QCNN) with minimal QPU downtime. Evaluated on quantum-accelerated image classifiers.
Transfer Learning Load Balancer [14]	Assigns varying quantum task sizes based on classical encoder output. Used in QNN transfer pipelines. Scheduler must match job to QPU capacity.
Modality-Driven Scheduler [15]	QPU resource allocation based on data modality (e.g., X-ray vs. MRI). Adjusts quantum job granularity at runtime. Improves training efficiency.
Lightweight QCNN Schedulers [13,40]	Partition and parallelize shallow quantum layers in hybrid QNNs. Supports multi-circuit execution per epoch.

**Table 7:** Real-QPU and hardware-in-the-loop evidence in the reviewed corpus. Reported metrics are not directly comparable because studies differ in backend, workload, circuit depth, and evaluation objective.

Study	Evaluation Setting	Workload Focus	Reported Metrics	Main Limitation
Giortamis et al. [23]	Cloud/QPU-oriented runtime evaluation	Quantum job multiplexing and scheduling	Waiting time, scheduling efficiency, fidelity-aware execution	Not evaluated as an end-to-end medical imaging pipeline
Murali et al. [28]	NISQ multi-programming/hardware-aware evaluation	Crosstalk and concurrent execution	Fidelity degradation, crosstalk behavior, execution reliability	Security and isolation implications are indirect for medical workflows
Liu et al. [24]	Quantum cloud mapping/hardware-aware simulation and profiling	Qubit mapping, error-aware allocation, multi-programming	Fidelity estimates, mapping quality, execution success indicators	Medical imaging and noisy-label retraining not directly evaluated
Landman et al. [11]	Medical QML evaluation with quantum-method benchmarking	Medical image classification	Classification performance and quantum-model behavior	Limited systems/runtime metrics such as queueing, latency, or deployment overhead

**Table 8:** Memory, encoding, and data movement mechanisms relevant to hybrid quantum-classical workflows (RQ2).

Technique	Description
Virtual Quantum Memory (Qoala) [30]	Maps logical qubit indices to physical hardware via OS-managed address spaces. Ensures multi-user isolation.
Topological Qubit Reuse [33]	Recycles qubits using circuit dependency graphs. Minimizes required hardware qubits.
Ancilla Reset and Uncompute [31,32]	Resets ancilla qubits during execution. Supports dynamic reuse and garbage collection.
Compiler-Informed Allocation [13]	Allocators use compiler hints for early qubit reuse. Supports shallow QCNN memory efficiency.
Noise-Aware Memory Selection [24]	OS selects qubits based on fidelity/health metrics. Avoids noisy memory blocks.
Cross-Memory Data Streaming [15,30]	Shared memory buffers between CPU and QPU reduce copy overhead.
Robust Training Buffers [42]	Label confidence storage, sample weights, and gradient tracking buffers increase memory usage in robust QNNs.
Hybrid Caching Techniques [43,47]	Pins noisy samples or key activations in RAM/GPU to avoid retraining I/O.
Modality-Aware Memory Design [15]	Optimizes QNN memory paths depending on data modality (e.g., 2D vs. 3D scans).
Lightweight Circuits [40,48]	Shallow quantum models minimize memory pressure but need more iterations. Suited for noisy-label datasets.

**Table 9:** Conventional distributed-systems controls versus quantum-specific controls in hybrid hospital-to-cloud pipelines.

Objective	Conventional Controls	Quantum-Specific Additions
Confidentiality	TLS, encryption at rest, IAM	circuit confidentiality, restricted backend telemetry, blind/obfuscated execution where available
Isolation	containers/VMs, network segmentation, RBAC	qubit partitioning, crosstalk-aware placement, temporal separation of sensitive jobs
Integrity	artifact signing, provenance, attestation	circuit-hash verification, calibration snapshotting, backend-selection provenance
Monitoring	audit logs, rate limits, SIEM	malicious-circuit detection, anomalous gate-pattern inspection, QPU telemetry monitoring

**Table 10:** Virtualization and containerization effects on reproducibility, performance, and deployment (RQ4).

Approach	Impact on Performance/Scalability
Qubernetes Containers [25]	Container-based scheduling using Kubernetes. Supports auto-scaling of QNN training. Seamless integration with cloud-native workloads.
cuQuantum in Docker [38]	Docker + cuQuantum accelerates simulation of large QNNs (e.g., QCNN). Achieves 10× throughput on multi-GPU clusters.
Hybrid Inference VM [15,39]	Container/VM hosting of quantum inference engines for medical diagnosis. Balances latency and flexibility across hardware.
Robust Training Containers [42,50]	Self-supervised QNNs trained under noise with dynamic checkpointing. Containers support rapid retraining.
Cross-Platform Reproducibility [43]	Containerized pipelines tested across edge/cloud environments for robustness evaluation. Ensures reproducibility and testing at scale.
Real-Time Feedback Overhead [48,51]	Identifies scheduling jitter in feedback-sensitive workloads. Requires container/OS tuning to meet latency targets.
Batching/Compilation Strategies [13]	Aggregates QNN circuit jobs into containers to reduce switching overhead. Supports microservice-style inference APIs.

**Table 11:** Isolation, security, and auditability considerations for hybrid quantum-classical workflows (RQ3).

Aspect	Details
Crosstalk and Side-Channels [34,35]	Adversarial circuits leak via crosstalk on shared QPU. OS must partition or schedule securely.
Qubit-Level Isolation [23,24]	QuCloud and QOS support job separation through qubit allocation. OS isolates coherence domains.
Blind Quantum Computing [41]	Entanglement-based privacy-preserving QNN execution. Prevents server from observing input/output.
Model Extraction Risks [7]	QNNs queried via API can be mimicked by attackers. OS may rate-limit or obfuscate response structure.
Anomaly Monitoring [49]	Runtime inspection of suspicious gate patterns, resource contention, and anomalous circuit behavior.
Audit Logs and Traceability [14]	Generate metadata: job IDs, hashes, timestamps. Log circuit-level events without raw data.
Containerized Execution [25]	IBM Qiskit Runtime and similar platforms sandbox user code. Prevent low-level hardware access.
Encryption of QNN Metadata [24,41]	Secure circuit parameters in-flight. Combine TLS and quantum-safe primitives.
Compliance Enforcement [14,49]	Integrate consent tracking and anonymization services. Align with medical privacy frameworks.

**Table 12:** Hybrid-specific design guidelines derived from the cross-RQ synthesis.

Guideline	Rationale	Evidence	Medical Imaging Implication
Treat QPU access as asynchronous and stateful	Queueing, calibration state, and backend availability can change between training and inference	Moderate	Log backend, calibration snapshot, and execution window for reproducibility
Track backend-selection provenance	Circuit retries across different QPUs or simulators can change fidelity and measurement statistics	Emerging	Audit diagnostic outputs with backend and retry metadata
Budget shots across retraining loops	Noisy-label correction, validation, and uncertainty estimation can multiply circuit evaluations	Moderate	Report shot count per image, per epoch, and per retraining stage
Treat encoding as a systems boundary	Image compression, patching, and quantum encoding dominate data movement and reproducibility	Strong	Preserve preprocessing, feature extraction, and encoding provenance
Use persistent or batched runtimes for VQA loops	Repeated classical–quantum roundtrips can dominate training time	Moderate	Prefer batching, caching, and persistent sessions during training/retraining
Separate cloud security from QPU-specific isolation	Standard access control does not address crosstalk or qubit co-location risks	Moderate	Combine healthcare compliance logs with QPU-aware placement records
Record stochastic compilation settings	Transpilation and mapping choices may affect circuit depth, fidelity, and repeatability	Emerging	Store circuit hashes, transpiler versions, seeds, and optimization levels

**Table 13:** Positioning of this review relative to recent surveys.

Review	Scope	Systems	Medical	Key Gap
Gupta et al. [19]	QML in digital health	Low	High	Focuses on applications; lacks scheduling, memory, and deployment analysis
Shahriyar & Tanbhir [20]	QML for medical imaging	Low	High	Provides taxonomy; no systems/runtime perspective
Idzikowski et al. [21]	QML in healthcare	Low	High	Reviews trends; lacks system-level design insights
**This review**	Hybrid quantum systems (systems level)	High	Moderate	Adds evidence maturity, system analysis, and design guidelines

## Data Availability

No new data were created or analyzed in this study. Data sharing is not applicable to this article.
